# Structure–Function Relationships in Geographic Atrophy Based on Mesopic Microperimetry, Fundus Autofluorescence, and Optical Coherence Tomography

**DOI:** 10.1167/tvst.14.2.7

**Published:** 2025-02-05

**Authors:** Souvick Mukherjee, Thilaka Arunachalam, Cameron Duic, Maria Abraham, Christine Orndahl, Supriya Menezes, Elvira Agrón, Maximilian Pfau, Tharindu de Silva, Clare Bailey, Alisa T. Thavikulwat, Sunil Bellur, SriniVas R. Sadda, Emily Y. Chew, Brett G. Jeffrey, Wai T. Wong, Tiarnan D. L. Keenan

**Affiliations:** 1 National Eye Institute, National Institutes of Health, Bethesda, MD, USA; 2 The Emmes Company, LLC, Rockville, MD, USA; 3 Institute of Molecular and Clinical Ophthalmology Basel, Basel, Switzerland; 4 Department of Ophthalmology, University Hospital Basel, Basel, Switzerland; 5 Bristol Eye Hospital, Bristol, UK; 6 Doheny Eye Institute, Pasadena, CA, USA; 7 University of California Los Angeles, Los Angeles, CA, USA; 8 Tiresias Biopharma Consulting LLC, Half Moon Bay, CA, USA

**Keywords:** microperimetry, structure–function relationships, geographic atrophy, age-related macular degeneration, chorodial hypertransmission

## Abstract

**Purpose:**

To examine relationships between retinal structure and visual function in geographic atrophy (GA) by analyzing spatial agreement between absolute scotomas and macular structure, focusing on (1) choroidal hypertransmission, a key feature of complete retinal pigment epithelium and outer retinal atrophy (cRORA), and (2) fundus autofluorescence (FAF)–defined GA.

**Methods:**

Mesopic microperimetry (using a novel T-shaped pattern) and multimodal imaging were recorded longitudinally in a phase II GA trial. Horizontal and vertical optical coherence tomography (OCT) line scans (corresponding to the T axes) were graded for choroidal hypertransmission; FAF images were graded for GA. Spatial concordance between zones of absolute scotoma and atrophy was quantified by the Dice similarity coefficient (DSC).

**Results:**

The analysis population comprised 24 participants (mean follow-up 26.8 months). For concordance between absolute scotoma and choroidal hypertransmission, estimated mean DSC was 0.70 (95% confidence interval [CI], 0.64–0.77). This was significantly higher than for FAF-defined GA (0.67; 95% CI, 0.61–0.74; estimated mean difference = 0.03, 95% CI, 0.02–0.05, *P* < 0.001). Mean OCT choroidal reflectivity was strongly associated with likelihood and severity of scotoma.

**Conclusions:**

Spatial concordance between absolute scotomas and GA structural features is moderately high and slightly higher for choroidal hypertransmission than FAF-defined GA. This supports choroidal hypertransmission, a key cRORA feature, as an outcome measure in interventional trials. OCT provides more information to explain visual function than FAF alone. However, given some discordance for both structural features, performing microperimetry alongside imaging remains important.

**Translational Relevance:**

These findings provide insights into the complex relationship between retinal structure and visual function and contribute to a nuanced understanding of outcome measures.

## Introduction

Geographic atrophy (GA) is the defining lesion of the atrophic subtype of late age-related macular degeneration (AMD).^1^ It affects approximately five million people worldwide and typically leads gradually to central vision loss to the level of legal blindness.^2^^–^^4^ GA is characterized by degeneration of the tissue complex comprising the retinal pigment epithelium (RPE), photoreceptors, and choriocapillaris. Macular areas affected by GA are expected to have profound vision loss in the corresponding visual field.^5^^,^^6^

A detailed understanding of the relationship between retinal structure and visual function is essential to understanding the pathophysiology of GA. However, our current knowledge of this complex relationship is incomplete. Retinal sensitivity can be measured with microperimetry, a visual function test that assesses sensitivity at specific retinal loci by using eye tracking to register the test pattern on a fundus image.^7^^–^^9^ Indeed, following the first use of microperimetry in GA,^10^ retinal sensitivity in the macula is now an important outcome measure in interventional trials aimed at slowing vision loss in GA since visual function in GA is typically captured poorly by common outcome measures like best-corrected visual acuity (BCVA).^11^^,^^12^ However, microperimetry is time-consuming to perform and requires a high degree of patient cooperation, while retinal imaging is more easily performed and provides objective outcomes. For these reasons, retinal imaging features are typically used in interventional clinical trials as surrogate outcomes for visual function. In particular, the rate of change in GA area on fundus autofluorescence (FAF) imaging was the primary outcome measure in recent phase III randomized controlled trials of complement inhibitors in GA,^13^^–^^15^ which supported the approval of these two drugs by the US Food and Drug Administration.^16^^,^^17^

The optical coherence tomography (OCT) definition of GA, as proposed by the Classification of Atrophy Meetings group, is complete RPE and outer retinal atrophy (cRORA).^18^ Choroidal hypertransmission, also known as a hypertransmission defect, is a key feature of cRORA and is the specific feature used to measure cRORA size. Hypertransmission defects that are large (i.e., at least 250 µm) have been shown to persist over time.^19^^,^^20^ Indeed, this structural feature has recently been proposed as a clinical trial endpoint for the ascertainment and measurement of GA, in the context of en face OCT imaging.^21^ Therefore, assessing the relative concordance of loci within GA lesions that are FAF-defined (as hypoautofluoresence) versus OCT-defined (as choroidal hypertransmission) with corresponding loci of decreased retinal sensitivity (as assessed by microperimetry) should provide insights into the utility of FAF versus OCT in the definition of structural GA lesions as an outcome measure.

We recently completed a phase II trial evaluating oral minocycline for GA.^22^ No significant treatment effect in slowing GA enlargement was detected, based on the primary outcome measure of FAF-defined GA area. In this trial, longitudinal mesopic microperimetry data were collected prospectively from all study eyes according to a prespecified protocol using a novel custom T-shaped testing grid. Multimodal imaging included OCT line scans spatially registered to the loci in the microperimetry testing grid. This data set provides an important opportunity to perform a detailed exploration of the relationship between retinal structure and visual function in eyes with GA. Specifically, the purpose of this analysis was to analyze the level of spatial concordance between zones with absolute scotomas on mesopic microperimetry and zones with GA defined by (1) choroidal hypertransmission on OCT or (2) hypoautofluorescence on FAF.

## Methods

### Study Procedures

The study design for the phase II trial evaluating minocycline for GA in AMD has been described previously.^22^ In brief, the study comprised a prospective, single-arm, phase II trial to evaluate the safety and possible efficacy of oral minocycline as a treatment to slow GA progression. The study was conducted at the National Institutes of Health (NIH) Clinical Center (Bethesda, MD, USA) and the Bristol Eye Hospital (Bristol, UK). The protocol was approved by the NIH Institutional Review Board and the South Central–Oxford B Research Ethics Committee and adhered to the tenets of the Declaration of Helsinki. Written informed consent was obtained from each participant before enrollment. The study was registered at www.clinicaltrials.gov (NCT02564978; registration date, October 1, 2015). Study oversight was provided by an independent external Data and Safety Monitoring Committee (DSMC) that approved the protocol prior to trial initiation and reviewed study data approximately every 6 months. This article relating to an exploratory outcome was not reviewed by the DSMC, according to the DSMC policy on review of manuscripts for exploratory outcomes.

Eligible participants were aged at least 55 years, with GA related to AMD in one or both eyes. If both eyes of an individual participant met the eligibility criteria, the eye with better BCVA was chosen as the study eye. At the eye level, the study eye eligibility criteria included (1) GA area >0.5 and <7.0 disc areas (approximately 1.0 to 17.78 mm^2^) on FAF, (2) BCVA Early Treatment Diabetic Retinopathy Study letter score ≥19 (Snellen 20/400), and (3) no current evidence or history of treatments for choroidal neovascularization. Eyes with or without GA foveal involvement were included. Further details of the eligibility criteria have been described previously.^22^

Enrolled participants were followed during an initial 9-month run-in phase without the administration of minocycline, with in-clinic assessments at baseline and months 3, 6, and 9. At month 9, the participants began taking oral minocycline 100 mg twice daily until study termination, with in-clinic assessments at months 12, 15, and every 6 months thereafter. The primary outcome measure was assessed at month 33 (i.e., after 24 months on minocycline), and the study ended at month 45.

**Figure 1.**
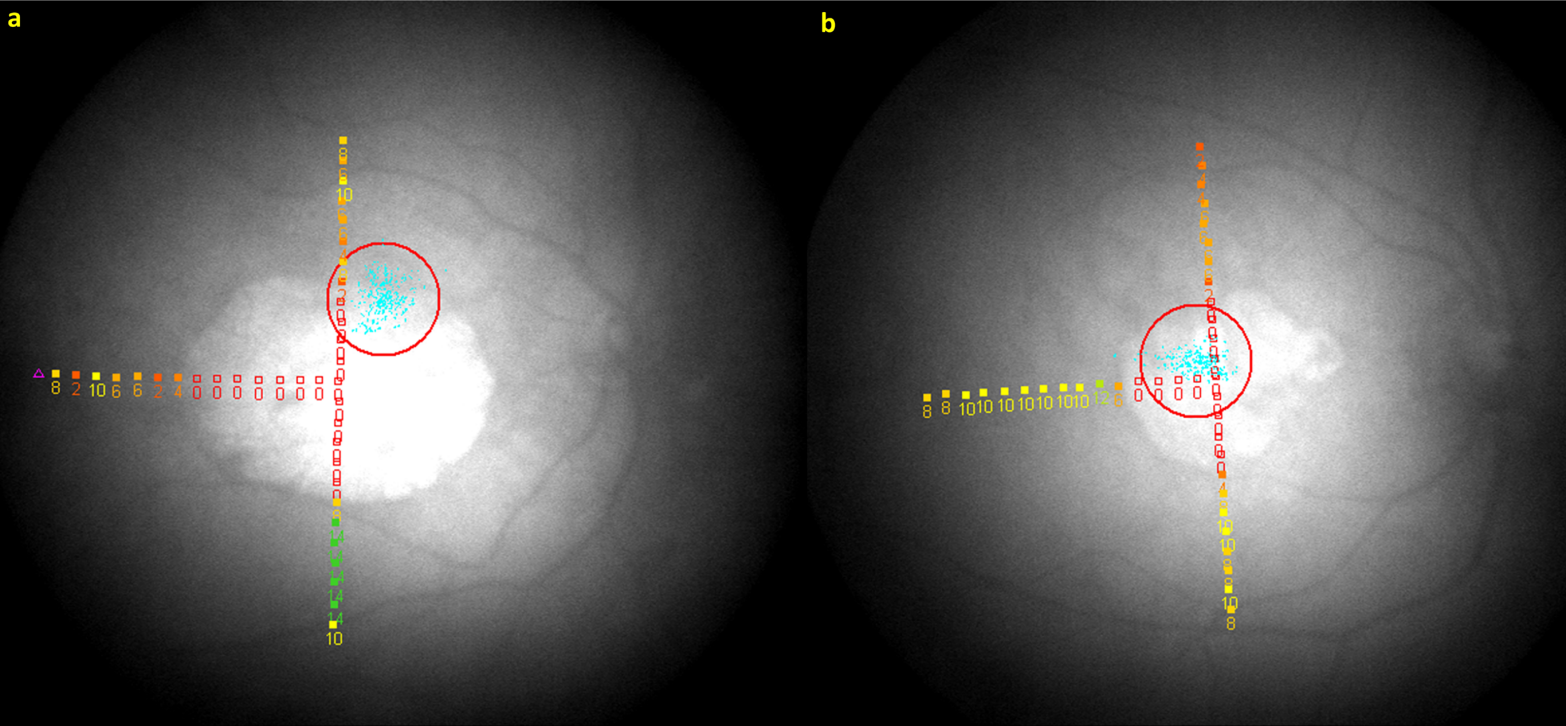
Images showing the results of mesopic microperimetry testing in two different study eyes (**a**, **b**) using the MP-1 microperimeter. The T-shaped testing grid is shown, consisting of 40 evenly spaced testing loci at 1° apart, extending from the anatomic fovea in three directions (15° temporally, 12° superiorly, and 12° inferiorly). The sensitivity results are shown numerically in dB (with absolute scotomas shown as *empty red squares*), together with the results of fixation testing (*blue dots*, with surrounding *red circle*). The test pattern in (**b**) appears skewed because the eye or head was slightly rotated during testing, but the eye-tracking and registration of the test pattern on a fundus image permit the test pattern to be placed consistently on the true anatomic horizontal and vertical axes, irrespective of eye rotation or positioning.

At baseline, month 3, and every 6 months thereafter, retinal sensitivity in the study eye was assessed by mesopic microperimetry, using the MP-1 microperimeter (Nidek Technologies, Padova, Italy). For each study eye, before the first test, the anatomic fovea was identified using spectral-domain OCT (Spectralis HRA + OCT; Heidelberg Engineering, Heidelberg, Germany), and a horizontal line scan passing through the foveal center was uploaded into the MP-1 software. Testing was performed using a novel custom T-shaped pattern, consisting of 40 evenly spaced testing loci with their center-points 1° apart (Fig. 1). The intersection of the T-shaped grid was placed at the anatomic fovea, with the three arms (or axes) of the T extending 15° temporally, 12° superiorly, and 12° inferiorly. A white Goldmann III stimulus (0.43° or 125 µm) was displayed for 200 ms at each testing locus. The stimulus intensities ranged from 127 to 1.27 cd/m^2^, which corresponded to macular sensitivities of 0 to 20 decibels (dB). The starting stimulus light attenuation was set at 10 dB, and a 4-2 staircase strategy was used. A red circle with a 3° radius was set as the fixation target on a white background luminance of 4 apostilbs (1.27 cd/m^2^). Following the first microperimetry test, all subsequent testing was performed in the instrument's “follow-up mode” to ensure spatial alignment of individual testing loci over longitudinal testing during the study. If participant cooperation was found to be unreliable or poor, the investigator had the discretion to stop testing according to the study protocol.

At each of these visits, multimodal imaging was performed according to a prespecified protocol. This comprised color fundus photography (Topcon 50-DX fundus camera [Topcon Medical Systems, Oakland, NJ, USA] using a MegaVision WS-5000 digital camera back [Santa Barbara, CA, USA]), with imaging field of 30° (2392 × 2014 pixels); short-wavelength “blue” FAF (Spectralis HRA2; Heidelberg Engineering), using wavelengths of 488 nm (excitation) and 500 nm (emission), captured in high-speed mode, with 15 averaged images, normalization on, and imaging field of 30° (768 × 768 pixels); and infrared reflectance and spectral-domain OCT (Spectralis HRA + OCT; Heidelberg Engineering), with the OCT captured in high-speed mode with enhanced depth imaging, automatic real time (ART) of five frames, and imaging field of 30° × 25°, comprising both (1) 121 horizontal B-scans with 60-µm spacing and (2) horizontal and vertical line scans through the foveal center (i.e., following the same horizontal and vertical lines as the T-shaped microperimetry testing grid) (Supplementary Fig. S1). The ART feature improves image quality by averaging multiple B-scans at each position to decrease noise.

### Fundus Autofluorescence Image Grading for Geographic Atrophy

The FAF images were graded by an external reading center (Doheny Image Reading and Research Lab, University of Southern California, Los Angeles) by two independent graders masked to participant identifiers, study visits, and microperimetry data. The grading was performed according to a standardized protocol, and the total area of definitely decreased autofluorescence was annotated and quantified by planimetry (Supplementary Fig. S2).^23^^,^^24^

### Optical Coherence Tomography Image Grading for Choroidal Hypertransmission

**Figure 2.**
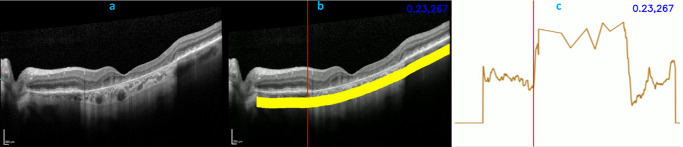
Grading of OCT line scans for choroidal hypertransmission. (**a**) A horizontal OCT line scan of a study eye with geographic atrophy is shown. (**b**) The scan has undergone automated segmentation to create a choroidal slab (*yellow*). (**c**) The results of reflectance profiling of the choroidal slab, where the intensity of the hypertransmission signal within the choroidal slab was quantified, averaged, and plotted graphically as a function of A-scan position. Together with qualitative inspection of the OCT scans, the hypertransmission reflectance plots were used to perform manual segmentation of spatial limits of the GA lesion on each OCT line scan (marked in *blue* as “OCT GA”). The numbers at the *top right* of (**b**) and (**c**) in *blue* show the normalized reflectance value of 0.23 (range, 0–1), from the 267th column of this B-scan.

The horizontal and vertical OCT line scans were evaluated and zones of choroidal hypertransmission marked by a trained grader (TA) under the supervision of a retina specialist (TK). Both individuals were masked to participant identifiers, study visit, and microperimetry data. Specifically, the OCT line scans underwent automated segmentation to detect Bruch's membrane, using a validated deep learning OCT segmentation model based on DeepLabv3+.^25^ On each A-scan, the mean hypertransmission signal intensity within the choroidal slab (defined as 64 to 200 µm beneath Bruch's membrane) was computed and graphically plotted as a function of A-scan position. For the choroidal slab, the offset of 64 µm was selected, in keeping with previous studies,^26^ to minimize the inclusion of large hyporeflective choroidal vessels that can interfere with the assessment of hypertransmission. Together with qualitative inspection of the OCT scans, these quantitative choroidal hypertransmission reflectance plots were used by the grader to manually segment the spatial limits of the GA lesion on the OCT B-scan (Fig. 2). In all cases, the zones of choroidal hypertransmission were representative of cRORA, since they were at least 250 µm in length and were accompanied by attenuation of the RPE and evidence of overlying photoreceptor degeneration.

### Overlaying of Sensitivity Measurements on Retinal Imaging

The sensitivity measurements from microperimetry were overlaid on the corresponding OCT line scans obtained at the same visit to enable spatial correspondence between microperimetry testing loci and segmented boundaries of structural GA lesions. Specifically, the sensitivity measurements from the horizontal line of the T-shaped testing grid were overlaid on the horizontal OCT line scan from the same visit, and a similar process was followed for the vertical line of the T-shaped grid and the vertical OCT line scan (based on known foveal locations and known grid spacings for both the microperimetry and OCT line scans). Zones with absolute scotomas on microperimetry (see Fig. 1) were marked on the OCT line scans as “−1” (denoting no response to the brightest 0-dB stimulus), whereas 0 dB indicates a response to the brightest stimulus. Using nearest neighbor interpolation, we obtained the linear length of absolute scotomas in the vertical and horizontal axes (Supplementary Fig. S3).

Separately, the zones of GA from FAF grading were also overlaid on the OCT line scans. Specifically, the GA contours from the FAF images were registered to the infrared reflectance images (acquired alongside the OCT line scans) using a previously validated registration algorithm.^27^

### Analyses of Spatial Concordance Between Absolute Scotomas and Macular Atrophy

The level of spatial concordance between retinal structure and retinal sensitivity was analyzed separately for the OCT and the FAF imaging. Specifically, the concordance between zones of absolute scotoma on the mesopic microperimetry and zones of choroidal hypertransmission (as representative of cRORA) on OCT was measured. Similarly, the concordance between zones of absolute scotoma and zones of GA on FAF was measured. In both cases, the level of concordance was quantified by the Dice similarity coefficient (DSC):

$$\begin{array}{c} DSC=\frac{2TP}{2TP+FP+FN} \end{array}$$

where true positive (TP) represents the number of pixels overlapping between zones of absolute scotoma and zones of atrophy on retinal imaging, false positive (FP) represents the number of pixels in zones of absolute scotoma without atrophy, and false negative (FN) represents the number of pixels in zones of atrophy without absolute scotoma. A DSC value of 0 signifies no overlap, while a DSC of 1 signifies complete overlap. A DSC above 0.7 is typically considered high, indicating substantial agreement, while a DSC below 0.4 is typically considered low.^28^ Importantly, however, these thresholds are somewhat arbitrary since the expected DSC depends strongly on the task and domain and is usually lower with smaller structures. For example, DSC values for the segmentation of areas or volumes are often higher than those for the segmentation of lines (as in this study). In previous ophthalmology studies, for the segmentation of retinal fluid areas on OCT B-scans, a DSC range of 0.72 to 0.78 was deemed to have high agreement.^29^ Similarly, for the automated segmentation of retinal fluid volumes on OCT cubes, a DSC range of 0.73 to 0.74 was considered high.^30^

**Figure 3.**
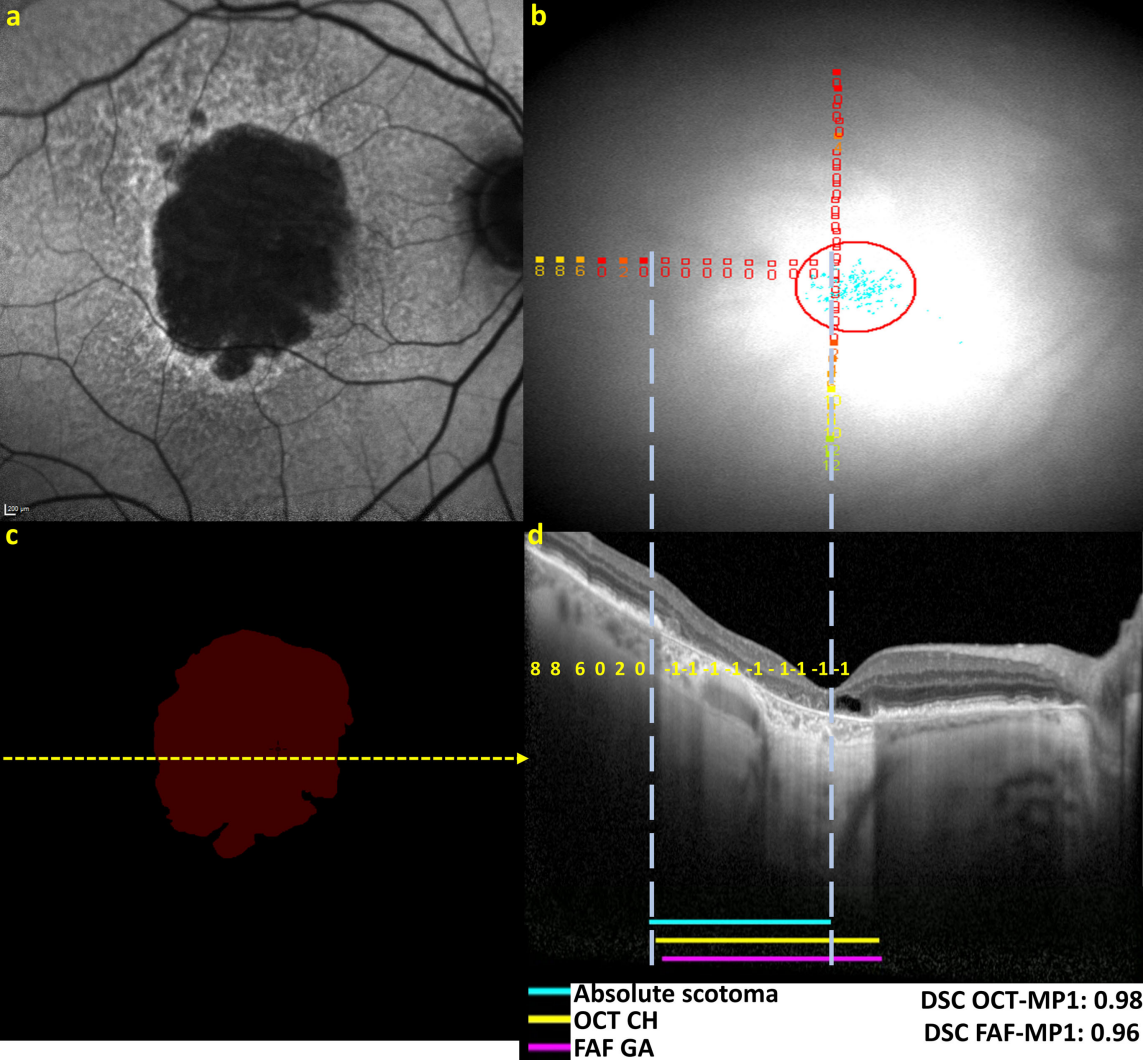
Retinal imaging, grading of geographic atrophy, overlaying of sensitivity measurements, and measurement of structure–function concordance for a study eye with GA. (**a**) Fundus autofluorescence imaging, with reading center segmentation of GA lesion shown in *red* in (**c**). (**b**) Microperimetry-derived retinal sensitivity measurements for the same GA lesion. (**d**) Horizontal optical coherence tomography line scan, corresponding to the *yellow dashed line* in (**c**) and the horizontal microperimetry testing line in (**b**). Images (**b**) and (**d**) have been aligned and scaled (with alignment of central foveal location shown by the *dashed white line* through the foveal center) for accurate alignment of structure and function measurements. In (**d**), the zone graded with choroidal hypertransmission is shown in *yellow* and labeled “OCT CH.” This zone can be spatially juxtaposed with the positions of microperimetry testing loci recording an absolute scotoma (*blue line* in **d** demarcated by *dashed vertical white lines*), as well as the spatial boundaries of the GA lesions identified using FAF (*purple zone* labeled “FAF GA”). The zone of choroidal hypertransmission in the temporal macula corresponds closely to the zone with absolute scotomas on the microperimetry test (*unfilled red squares* in **b**, with close correspondence shown by *dashed white lines*). The DSCs relating the position of the absolute scotoma to OCT-identified GA and to FAF-identified GA for this example are listed as “DSC OCT-MP1” and “DSC FAF-MP1,” respectively (*lower right*).

The complete workflow is summarized in Figure 3 by a representative example.

To decrease potential heterogeneity in data from differences in technician procedures during microperimetry testing at different sites, only data from the NIH site were used, while data from the Bristol Eye Hospital site (*n* = 7 participants) were excluded.

To evaluate whether there was a higher concordance between structure and function for (1) GA grading on FAF images versus (2) choroidal hypertransmission on OCT images, the DSCs for (1) and (2) with absolute scotomas on microperimetry were compared using a linear mixed-model analysis with random effects accounting for the correlation between participants’ horizontal and vertical scans across visits. The covariance structure “autoregressive” (AR(1)) was chosen for the random effects, as it resulted in the best model fit (i.e., the lowest Akaike's information criterion [AIC]). Hence, all available time points were included in the analyses, rather than a single time point per eye, with the random effects and associated covariance structure accounting for correlation between observations at different time points of the same eye. Results were considered significant if the *P* value was <0.05 (two-sided test).

As sensitivity analyses, these analyses were repeated with the exclusion of any OCT line scans with only one absolute scotoma point since, in those cases, the degree of concordance as measured by DSC was expected to be relatively low, thereby impacting the overall DSC results. The covariance structure “heterogeneous autoregressive” (ARH(1)) was chosen for the random effects, as it resulted in the best model fit in these analyses.

### Analyses of Relationship Between Choroidal Reflectance and Likelihood of Scotoma

In further analyses that pooled all microperimetry tests, the microperimetry test loci were classified into three groups, based on their sensitivity measurements, in accordance with the literature^31^^,^^32^: (1) absolute scotomas (sensitivity below 0 dB), (2) relative scotoma (sensitivity from 0 to 8 dB), and (3) nonscotomatous loci (sensitivity greater than 8 dB). A histogram was plotted to study the relationship between mean choroidal OCT reflectivity at that microperimetry test locus and likelihood of scotoma at that locus. In order to evaluate whether the choroidal reflectivity values significantly differed between the three groups of loci (i.e., absolute scotomas, relative scotomas, and nonscotomatous loci), a linear mixed-model analysis with random effects was performed accounting for the correlation between loci of participants’ A- and B-scans across visits. The covariance structure “heterogenous compound symmetry” was chosen for the random effects, as it resulted in the best model fit (i.e., the lowest AIC). A global *F*-test was performed to determine if there were significant differences between any of the loci classifications (i.e., absolute scotomas versus relative scotomas versus nonscotomatous loci). If the global *F*-test was significant, pairwise tests were performed to assess for differences between each pair of loci classifications. Results were considered significant if the *P* value was <0.05 (two-sided test).

### Qualitative Analyses of Scans With Low Concordance Between Retinal Structure and Visual Function

All scans with a DSC value lower than 0.5, indicating low concordance between zone of absolute scotoma and zone of atrophy (either FAF-defined GA or choroidal hypertransmission), were evaluated qualitatively by a retina specialist (TK). The main reason for low concordance was recorded.

## Results

Of the 30 participants from the NIH site, 6 were excluded owing to the imaging data being either missing or not captured in follow-up mode. Therefore, the analysis population comprised 24 participants. The baseline characteristics of the study population are shown in the Table. Mean (standard deviation [SD]) age was 74.3 (7.8) years (range, 63–89 years). Mean (SD) follow-up, based on microperimetry testing, was 26.8 (11.4) months (range, 3–51 months). The total number of microperimetry tests was 117, which corresponded to a mean (SD) of 4.9 (1.7) visits per participant (range, 2–8 visits).

**Table.** Participant Demographic and Ocular Characteristics at Study Baseline

Variable	Characteristic or Statistic	Value
	*N*	24
Sex, *n* (%)	Male	7 (29)
	Female	17 (71)
Age	Mean (SD; range)	74.3 (7.8; 63–89)
Ethnicity, *n* (%)	Hispanic or Latino	2 (8)
	Not Hispanic or Latino	21 (88)
	Unknown	1 (4)
Race, *n* (%)	Asian	2 (8)
	Black	1 (4)
	White	21 (88)
Fovea involving GA based on FAF (study eye), *n* (%)	Subfoveal	14 (58)
	Not subfoveal	10 (42)
Square root of GA area based on FAF (study eye), mm	Mean (SD)	2.6 (0.9)
BCVA letter score; Snellen distance,^a^ (study eye)	Mean (SD)	71.1 (15.7); 56.8 (72.6)
LLVA letter score; Snellen distance,^a^ (study eye)	Mean (SD)	54.1 (16.3); 118.2 (108.7)

Denominators are the number of participants with successful overlay of microperimetry and OCT data (*N* = 24). Percentages are rounded to the nearest whole number. LLVA, low-luminance visual acuity.

a Snellen distance represents the denominator in the 20/X Snellen notation (i.e., the size of letters read on a Snellen visual acuity chart at 20 feet); for <20/800 Snellen values, 800 was used as the Snellen distance.

### Spatial Concordance Between Absolute Scotomas and Macular Atrophy

For each horizontal and vertical OCT line scan, the DSC was calculated to quantify the level of concordance between zones of absolute scotoma and zones of choroidal hypertransmission. In the whole analysis population, across all horizontal and vertical scans and all study visits, the estimated mean DSC from the mixed-effects model was 0.70 (95% confidence interval [CI], 0.64–0.77). Similarly, for the concordance between absolute scotomas and FAF-defined GA, the estimated mean DSC was 0.67 (95% CI, 0.61–0.74). The DSC was significantly higher for the OCT grading than for the FAF grading (estimated mean difference = 0.03; 95% CI, 0.02–0.05; *P* < .001).

The analyses were repeated in a modified data set that excluded the 12 test lines with only one absolute scotoma. For the concordance between absolute scotomas and choroidal hypertransmission on OCT, the estimated mean DSC from the mixed-effects model was 0.74 (95% CI, 0.10–1.38). Similarly, for the concordance between absolute scotomas and FAF-defined GA, the estimated mean DSC was 0.71 (95% CI, 0.03–1.39). Again, the DSC was significantly higher for the OCT grading than for the FAF grading (estimated mean difference = 0.03; 95% CI, 0.02–0.05; *P* < 0.001).

### Relationship Between Choroidal Reflectance and Likelihood of Scotoma

**Figure 4.**
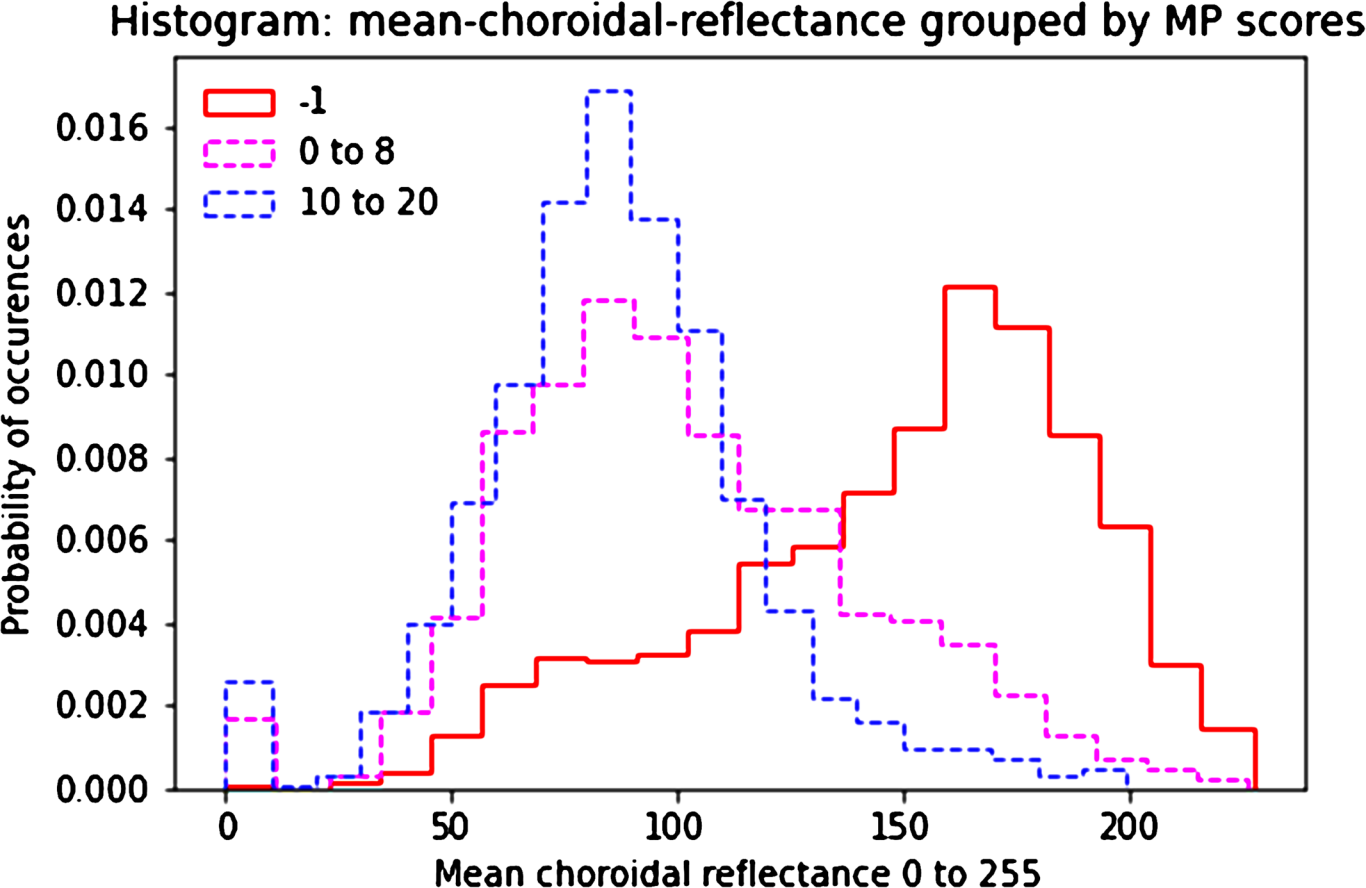
Histogram showing the relationship between mean choroidal reflectance and probability of scotoma occurrence and type at that location. The *solid red line* indicates testing loci with absolute scotomas, the *dashed pink line* indicates testing loci with relative scotomas, and the *dashed blue line* indicates nonscotomatous testing loci.

The mean (SD) grayscale reflectivity value (range, 0–255) in the choroidal OCT slab, across the whole study population of horizontal and vertical OCT lines, was 112.5 (46.2) arbitrary units. The microperimetry test loci were classified into absolute scotomas, relative scotomas, and nonscotomatous loci. The estimated mean grayscale reflectivity values in the choroidal OCT slabs based on the mixed-effects model described previously, according to the three retinal sensitivity groups, were 128.14 (95% CI, 125.36–130.93) for the absolute scotomas, 113.19 (95% CI, 110.50–115.88) for the relative scotomas, and 103.80 (95% CI, 101.01–106.59) for the nonscotomatous loci. Figure 4 shows a histogram of the relationship between mean choroidal OCT reflectivity and likelihood of scotoma. Given a significant difference was observed between at least two of the loci groups (*F*(2, 3632) = 164.41; *P* < 0.001), pairwise tests were performed between each pair of loci groups. The choroidal reflectivity values differed significantly between each pair of loci groups: grayscale reflectivity in absolute scotomas was significantly higher than in nonscotomatous loci (estimated mean difference = 24.34; 95% CI, 21.69–26.99; *P* < 0.001) and relative scotomas (estimated mean difference = 14.95; 95% CI, 12.73–17.18; *P* < 0.001), and grayscale reflectivity in relative scotomas was significantly higher than in nonscotomatous loci (estimated mean difference = 9.39; 95% CI, 7.30–11.47; *P* < 0.001).

### Qualitative Analyses of Scans With Low Concordance Between Retinal Structure and Visual Function

**Figure 5.**
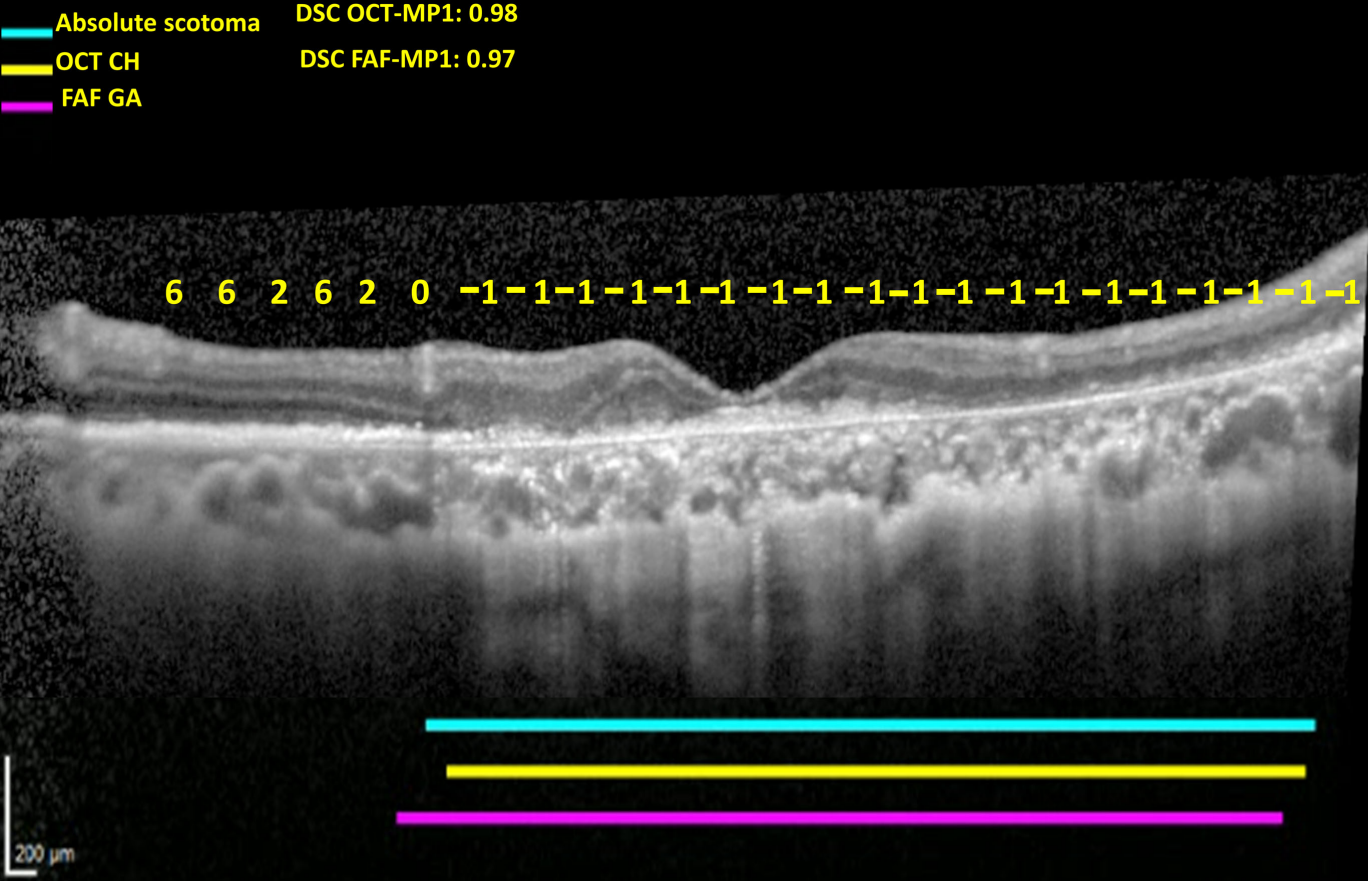
Vertical OCT line scan of a study eye with GA, with overlaying of sensitivity measurements from microperimetry testing at the same visit (with *yellow numbers* indicating dB measurements and −1 indicating absolute scotomas). The sensitivity values, shown in *yellow*, are positioned over their corresponding test locations. The *solid blue line* indicates regions of absolute scotomas from microperimetry testing. The *solid yellow line* indicates regions with choroidal hypertransmission on OCT (CH). The *solid pink line* indicates regions with GA on the corresponding FAF image. The DSC values are shown.

Figure 5 demonstrates an example of a vertical OCT line scan with a high level of concordance between zones of absolute scotoma on microperimetry testing and both (1) zones graded with choroidal hypertransmission on OCT (i.e., representative of cRORA) and (2) zones graded with GA on corresponding FAF imaging.

For the concordance between absolute scotomas and choroidal hypertransmission on OCT (and/or FAF-defined GA), all instances with a DSC value lower than 0.5 were evaluated qualitatively by a retina specialist (TK). Two main situations were encountered, comprising (1) cases with an absolute scotoma present despite the absence of hypertransmission (i.e., retinal sensitivity worse than predicted from retinal structure) and (2) cases with hypertransmission present despite the absence of an absolute scotoma (i.e., retinal structure worse than predicted from retinal function). For choroidal hypertransmission on OCT, of the 199 instances that comprised the OCT-microperimetry (MP) analysis, there were 35 instances with a DSC <0.5. Of these 35 instances, there were 17 instances with choroidal hypertransmission present despite the absence of an absolute scotoma at that location and 7 instances with an absolute scotoma present despite the absence of hypertransmission at that location. For FAF-defined GA, of the 199 instances that comprised the FAF-MP analysis, there were 42 instances with a DSC <0.5. Of these 42 instances, there were 20 instances with GA present despite the absence of an absolute scotoma at that location and 15 instances with an absolute scotoma present despite the absence of GA at that location.

**Figure 6.**
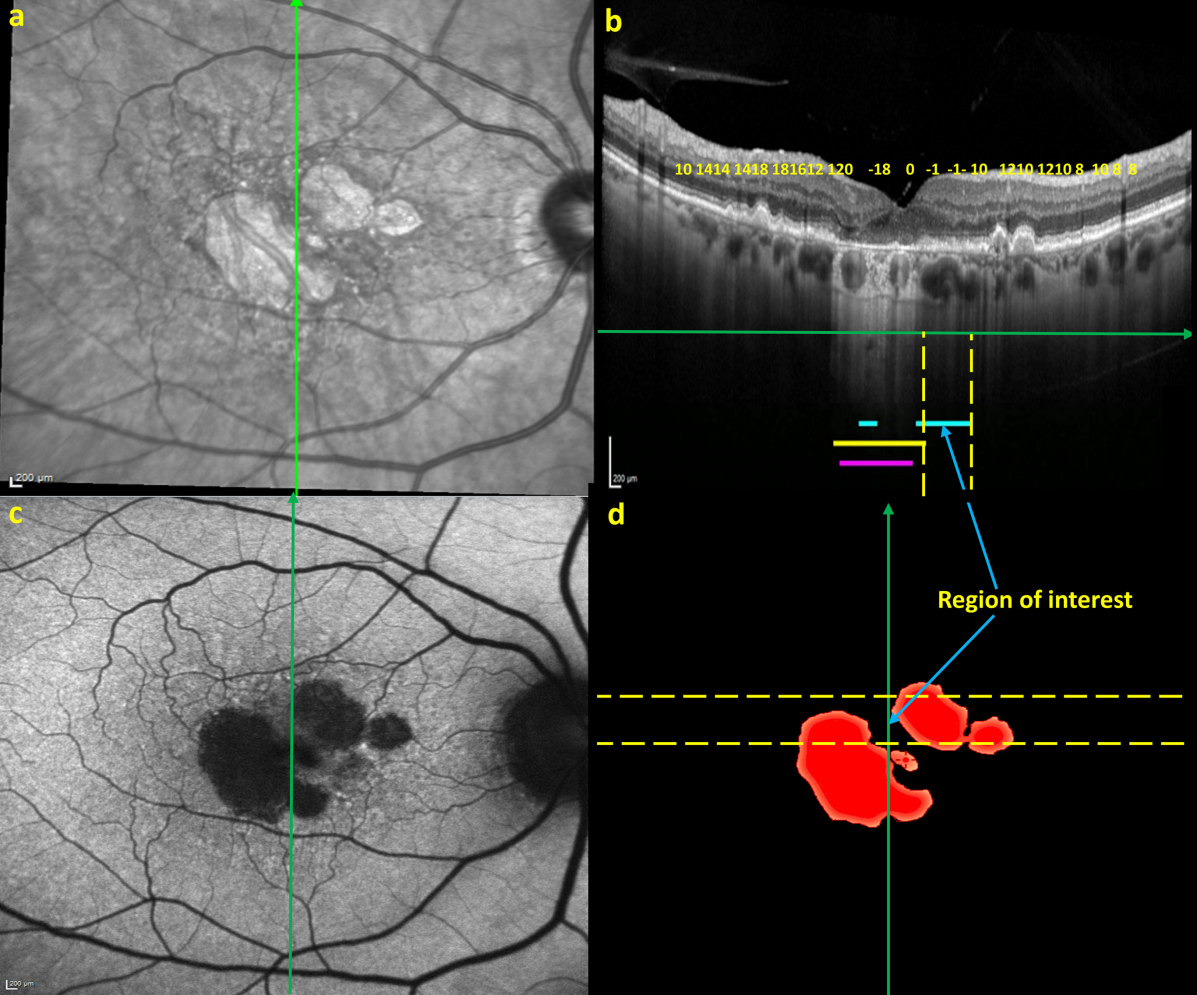
Representative case demonstrating the presence of an absolute scotoma despite the absence of choroidal hypertransmission on OCT. (**a**) Infrared reflectance and (**c**) FAF imaging showing areas with GA, with the corresponding GA contour from the FAF image shown in *red* in (**d**). (**b**) Vertical OCT line scan with sensitivity measurements overlaid. The *blue line* indicates regions of absolute scotomas, the *yellow line* indicates regions with choroidal hypertransmission, and the *pink line* indicates regions with GA on the corresponding FAF image. The corresponding OCT and microperimetry testing lines (*green arrows*) are observed to run between two GA lesions, just superior to the fovea. Absolute scotomas (marked in *yellow* as −1 in **b**) are present at this location, despite the absence of choroidal hypertransmission. This region of interest is shown in (**b**) and (**d**). As a result, the Dice similarity coefficient values in this case were low, at 0.34 (OCT) and 0.25 (FAF).

**Figure 7.**
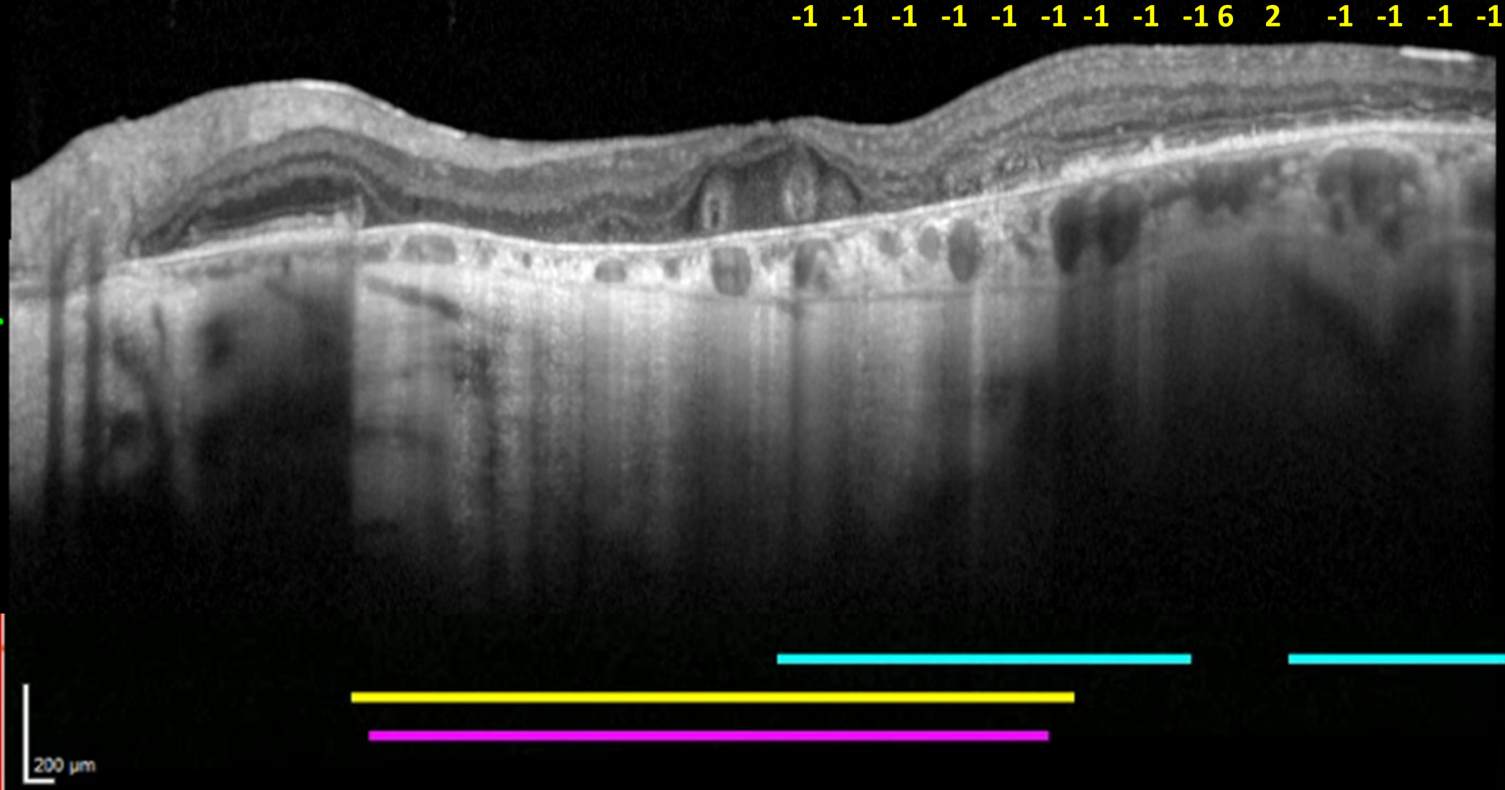
Representative case demonstrating, in the far temporal macula, the presence of absolute scotomas despite the absence of choroidal hypertransmission on this horizontal optical coherence tomography line scan with overlaying of sensitivity measurements. The *blue line* indicates regions of absolute scotomas, the *yellow line* indicates regions with choroidal hypertransmission, and the *pink line* indicates regions with GA on the corresponding FAF image. In the far temporal macula, absolute scotomas (marked in *yellow* as −1) are present, despite the absence of choroidal hypertransmission. Notably, this zone has pronounced outer retinal atrophy in the context of reticular pseudodrusen. As a result, the overall Dice similarity coefficients for this case were 0.64 (OCT) and 0.60 (FAF).

For the first situation, with an absolute scotoma present despite the absence of hypertransmission, two main explanations were observed. First, in some cases, the test line (i.e., the OCT line scan and corresponding microperimetry test line) ran between two GA lesions, such that hypertransmission was not present at that location on that particular line scan but was present in neighboring or nearby line scans (Fig. 6). Second, in other cases, an absolute scotoma was present in a zone of isolated outer retinal atrophy (i.e., without choroidal hypertransmission), particularly in the context of reticular pseudodrusen (as demonstrated in Fig. 7).

**Figure 8.**
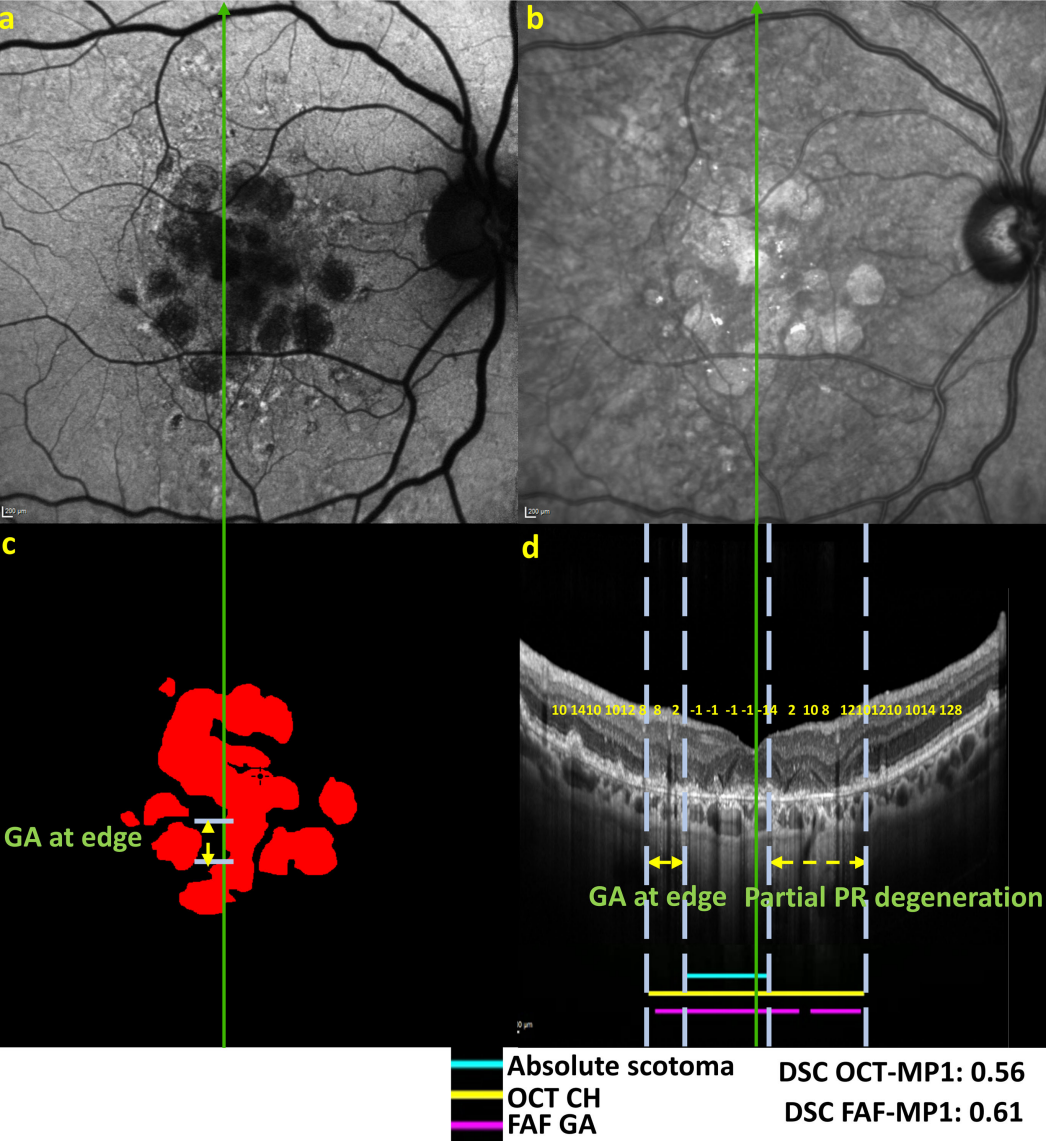
Representative case demonstrating the presence of choroidal hypertransmission on OCT and/or GA on fundus autofluorescence despite the absence of absolute scotomas. (**a**) Fundus autofluorescence imaging, with reading center grading of geographic atrophy shown in *red* in (**c**). (**b**) Infrared reflectance image. (**d**) Vertical optical coherence tomography line scan, corresponding to the *green vertical testing lines* in (**a**), (**b**), and (**c**). All images have been aligned and scaled, with alignment of central foveal location shown by the *green line* through the fovea. The DSC values are reported.

For the opposite situation, that is, with choroidal hypertransmission (and/or FAF-defined GA) present despite the absence of absolute scotomas, again, several explanations were observed. First, in some cases, the testing line was located at the edge of a GA lesion, such that choroidal hypertransmission (and/or FAF-defined GA) was present at that location but was absent in nearby line scans (as observed in Figs. 8c, 8d and region labeled by “GA at edge”). Second, in other cases, a zone of hypertransmission was present and the criteria of cRORA were met, but the degree of overlying photoreceptor degeneration may have been relatively lower at some locations (as seen in Fig. 8d for the region labeled “Partial photoreceptor (PR) degeneration”). In other cases, a relative scotoma occurred in the middle of a GA lesion (with hypertransmission and outer retinal atrophy both present on OCT imaging), with no apparent reason for the scotoma being relative but not absolute.

## Discussion

### Main Findings, Interpretation, and Implications

This study evaluated levels of spatial concordance between absolute scotomas on mesopic microperimetry and atrophy defined independently on two different imaging modalities in a clinical trial setting. The concordance between zones with absolute scotomas and zones with FAF-defined GA or choroidal hypertransmission on OCT was moderately high. The level of concordance was significantly higher with choroidal hypertransmission than with FAF-defined GA, although the difference was relatively small and might not be clinically meaningful.

These findings lend support to the use of FAF-defined GA as an outcome measure in natural history studies or interventional trials aimed at slowing GA progression (i.e., as a surrogate outcome measure for visual function). Importantly, however, this study demonstrates that the concordance is not perfect. Hence, an intervention might slow decline in visual function either more or less than that suggested by FAF-defined GA alone. For example, in the case of progression to isolated outer retinal atrophy, the decline in visual function could be more than that suggested by FAF-defined GA alone. Conversely, in the case of photoreceptor atrophy lagging behind RPE atrophy, the decline in visual function could be less than suggested by FAF-defined GA alone. For these reasons, we believe it remains important to perform microperimetry alongside FAF imaging in natural history studies and clinical trials. This is particularly true if a study drug's mechanism of action is expected to target photoreceptors more than RPE cells^33^ or if there is concern that a study drug may lead to a partial breakdown in the usual relationship between autofluorescence levels and visual function.^34^

The findings also lend support to the potential use of choroidal hypertransmission on OCT (a component feature of cRORA) as a quantitative outcome measure in interventional trials, as proposed recently for en face OCT,^21^ since the mean level of concordance was higher than for FAF-defined GA. Again, though, some important causes of discordance were observed, where an intervention might slow decline in visual function either more or less than that suggested by choroidal hypertransmission alone. In addition, in the scenarios mentioned above (if a study drug's mechanism of action is expected to target photoreceptors more than RPE cells or may lead to a partial breakdown in the usual relationship between autofluorescence levels and visual function), choroidal hypertransmission will not be very informative. However, one advantage of OCT over FAF alone is the more detailed three-dimensional information captured by OCT. In particular, this three-dimensional information allows a more granular evaluation of degeneration for the RPE and photoreceptor layers, considered separately; this appears highly relevant for many instances of discordance between structure and function demonstrated in the current study. For these reasons, we believe it is important to perform OCT imaging alongside FAF in natural history studies and interventional trials. Ideally, this should be accompanied by more sophisticated analyses of retinal structure than overall thickness alone. For example, in macular telangiectasia type 2, the primary outcome measure in two recent phase III studies was change over time in area of ellipsoid zone loss on OCT.^35^^,^^36^

The quantification of GA from FAF images is well established in the reading center setting. Typically, graders draw contours around areas of definitely decreased autofluorescence by planimetry, and the total area is calculated by automated software.^24^ This approach is considered relatively straightforward since the GA can be detected on a single two-dimensional image, and the difference in signal strength between areas with and without GA is typically high.

The quantification of GA from OCT images is a more recent development. The grading and measurement of choroidal hypertransmission (as the key feature of cRORA and the specific feature used to measure cRORA size) can be performed with relative ease on individual B-scans; however, this process is time-consuming, since the grading needs to be repeated on a large number of B-scans. For this reason, en face OCT imaging using closely spaced raster B-scans has been proposed.^21^ This has the advantage of condensing the three-dimensional OCT information from a desired retinal layer into a single two-dimensional image, so that choroidal hypertransmission can be graded by contouring and area measurement (i.e., similar to the process used for FAF images). This approach sometimes requires manual correction of segmentation errors and has been proposed and validated mostly using swept-source OCT devices (capable of rapidly acquiring many closely spaced raster B-scans), which are less widely available than spectral-domain devices.^21^ However, spectral-domain OCT devices are also able to generate en face images and may be sufficient.^37^

### Discordance Between Retinal Structure and Visual Function

The moderately high level of concordance between absolute scotomas and structural features of atrophy reflected many instances of high concordance (as demonstrated in Fig. 5), together with some instances of lower concordance (as demonstrated in Fig. 6b). Qualitative evaluation of instances with lower concordance demonstrated examples of discordance in both directions (i.e., scotomas without atrophy and atrophy without scotomas) and for different reasons.

One important phenomenon relates to complex situations where degeneration of the RPE and photoreceptor layers are not present simultaneously or to the same degree. In some instances of isolated outer retinal atrophy, absolute scotomas were present without choroidal hypertransmission and/or FAF-defined GA. Conversely, in some instances of RPE atrophy occurring disproportionately to photoreceptor degeneration, the presence of choroidal hypertransmission and/or FAF-defined GA in zones without absolute scotomas was likely related to the absence of total photoreceptor degeneration.

A second phenomenon relates to spatial considerations regarding differences between linear versus area-based assessments. Microperimetry involves area-based testing of visual function, since even small testing stimuli are presented over a certain area of retinal tissue and corresponding visual field, rather than at an individual pixel or cell. In this study, all loci were based on a Goldmann III size stimulus, which represents 0.43° of the visual field or 125 µm diameter of retinal tissue. By contrast, the OCT line scans (and the FAF-defined GA contours overlaid on them) represent linear assessments. Hence, in some instances where choroidal hypertransmission and/or FAF-defined GA was present without absolute scotomas, the likely explanation was that the testing line was just inside a GA lesion (as demonstrated in Fig. 8a). With a microperimetry locus partly inside but partly outside an area of atrophy, sufficient stimulation of the retinal tissue just outside the atrophic area might be sufficient to avoid an absolute scotoma. Conversely, in some instances where absolute scotomas were present without choroidal hypertransmission and/or FAF-defined GA, the likely explanation was that the testing line was just outside a GA lesion or even just between two closely spaced GA lesions (as demonstrated in Fig. 6). In this case, if most of the microperimetry locus fell on atrophic tissue, the stimulation might be insufficient to register a response, despite the apparent absence of atrophy at the central line itself.

### Testing Approach and Algorithmic Workflow

The testing approach and algorithmic workflow developed in this study may be helpful in other studies. The main advantage of having linear microperimetry testing exactly registered with OCT line scans is that overlaying the sensitivity values onto the OCT scans permits detailed comparison of three-dimensional retinal structures with visual function at specific locations, including independent evaluations of the RPE and photoreceptor layers. The workflow also included computer-assisted grading of choroidal hypertransmission, permitted by automated retinal segmentation^21^ and creation of choroidal slabs, together with automated OCT reflectivity profiling. In addition, comparison between concordance with OCT and with FAF was permitted by overlaying the FAF-defined GA contours onto the same microperimetry testing lines through automated registration between FAF and infrared reflectance (IR)/OCT.^22^

This automated approach enabled a large-scale analysis of the relationship between OCT choroidal reflectance and likelihood of scotoma, which showed that choroidal reflectance is extremely strongly associated with the likelihood of scotoma presence and degree. However, the degree of overlap between the three histograms in Figure 7 shows that absolute levels of choroidal reflectance by themselves are not sufficient to predict scotoma presence and degree with complete certainty.

### Comparison With Literature

Multiple previous studies have demonstrated an association between increasing GA area and worsening of retinal sensitivity, or increasing number of scotomatous points, on microperimetry.^38^^,^^39^ However, more granular studies are required that can explore structure–function relationships by analyzing regions of concordance and discordance spatially, ideally with GA defined on different imaging modalities. Some previous studies have explored relationships between en face OCT images (with GA segmentation) and microperimetry sensitivity maps. In one study, retinal sensitivity was observed to decrease precipitously at the GA margins, with sensitivity values in most of the GA junctional zone appearing similar to those of distal regions without GA.^40^ However, unlike our study, no analyses of three-dimensional OCT features like choroidal hypertransmission were made, and no FAF imaging was reported.

Other studies have overlaid microperimetry maps onto corresponding FAF images and OCT scans, similar to our approach. For example, one study observed that ellipsoid zone absence at baseline was associated with higher risk of subsequent development of a dense scotoma at that point, although this study was limited by a small sample size of seven eyes.^41^ Another study overlaid sensitivity maps on OCT scans of eyes with GA and observed a strong association between increasing number of structural features on OCT and lower sensitivity.^42^ The strongest associations with absolute scotoma were found for abnormalities of the RPE and external limiting membrane. It was also shown that OCT data, in combination with machine learning algorithms, can be used to infer pointwise retinal sensitivity with a mean absolute error of ±4.64 dB.^43^ However, unlike our study, no quantitative analysis of the spatial concordance (or reasons for local discordance) between structure and function was performed, and FAF imaging was not reported. Another study found that macular areas with nascent GA tended to exhibit sensitivity levels that were intermediate between those of areas with GA and without atrophy.^44^

Overall, over the past 20 years, microperimetry has been used widely to identify functional vision impairments in different retinal conditions, including AMD. However, lack of agreement on optimal testing approaches, together with the burdens of testing at scale, may have hindered its more widespread use as a viable clinical trial endpoint for GA.^45^ In this study, we provide quantitative analysis of its concordance with the clinical trial endpoint of FAF-defined GA and explore reasons for any discordance. We also investigate its concordance with another important biomarker and potential clinical trial endpoint, choroidal hypertransmission.

### Strengths and Limitations

The strengths of this study include its clinical trial setting, where microperimetry and multimodal imaging were performed prospectively at the same time points according to prespecified protocols. The direct alignment between the lines of the microperimetry testing and the OCT line scans enabled direct comparisons to be made between retinal structure and visual function. Similarly, direct comparisons were possible with FAF imaging, owing to registration between FAF and OCT images. Additional strengths include the relatively large amount of data, independent reading center grading of GA on FAF imaging, and the use of an automated algorithm to generate choroidal slabs on OCT, followed by automated reflectance profiling to quantify reflectance, to assist with manual grading of choroidal hypertransmission.

Potential limitations of the study include the interventional nature of the clinical trial. However, analyses of both structural and functional outcome measures have demonstrated no significant effect of the study drug, such that structure–function analyses should not be substantially affected by the pooling of study visits before and after study drug initiation. Other potential limitations include the exclusion of data from a small number of participants owing to appropriate multimodal imaging being unavailable. In addition, one potential limitation is the exclusion of data from the second site. This approach was decided before any data analysis to ensure a relatively homogeneous data set in which all microperimetry was performed by two ophthalmic technicians highly experienced in this testing. Given the much larger size of the NEI versus the Bristol microperimetry data set (with 117 vs. 21 separate tests, respectively), we do not believe that exclusion of the latter will have substantially affected the results. However, we recommend future studies at multiple sites toward potential replication of these findings. Finally, the study was not powered for these analyses, and as such, the results of these exploratory analyses should be interpreted with caution.

## Conclusions

In GA secondary to AMD, the spatial concordance between zones with absolute scotomas on mesopic microperimetry and zones with FAF-defined GA is moderately high but falls short of perfect. The same is true for spatial concordance between zones with absolute scotomas and zones with choroidal hypertransmission on OCT, as representative of cRORA. Comparison of the two demonstrates that concordance is higher for choroidal hypertransmission than for FAF-defined GA, although the small difference might not be clinically meaningful. As explanations for imperfect concordance, discordance between macular structure and function occurred in both directions (i.e., some cases with absolute scotomas despite no choroidal hypertransmission or GA and other cases with choroidal hypertransmission or GA but no absolute scotomas). Reasons for these included absolute scotomas from isolated outer retinal atrophy and absent scotomas from choroidal hypertransmission with only partial photoreceptor degeneration. Overall, concordance of absolute scotomas with choroidal hypertransmission was slightly superior to that with FAF-defined GA; in addition, OCT may provide more detailed information to explain visual function than FAF alone, particularly in cases where atrophy of the RPE and photoreceptor layers are not present simultaneously or to the same degree. These findings provide insights into the complex relationships between retinal structure and visual function in GA toward a nuanced understanding of structural outcome measures in interventional trials.

## Supplementary Material

Supplement 1

Supplement 2

Supplement 3
