# Supplementary material for: Structure–Function Relationships in Geographic Atrophy Based on Mesopic Microperimetry, Fundus Autofluorescence, and Optical Coherence Tomography
Source: Transl Vis Sci Technol. 2025 Feb 5;14(2):7. doi: 10.1167/tvst.14.2.7 (PMC11806430; doi:10.1167/tvst.14.2.7)

Supplementary Figure 1. Infrared reflectance (A and C) and optical coherence tomography (OCT; B and D) imaging of a study eye with geographic atrophy. (B) shows the horizontal OCT line scan through the foveal center, while (D) shows the vertical OCT line scan through the foveal center. These horizontal and vertical OCT line scans correspond exactly to the horizontal and vertical lines of the T-shaped microperimetry testing grid.

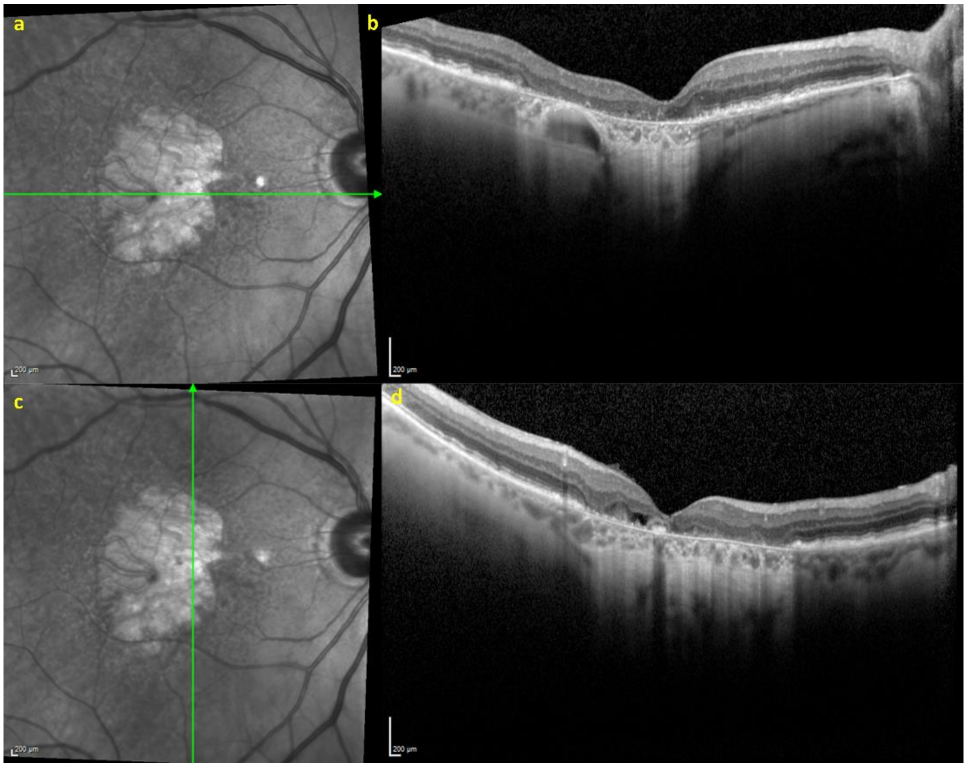

Supplement: Supplement 1 [file tvst-14-2-7_s001.pdf]
