# Supplementary material for: Structure–Function Relationships in Geographic Atrophy Based on Mesopic Microperimetry, Fundus Autofluorescence, and Optical Coherence Tomography
Source: Transl Vis Sci Technol. 2025 Feb 5;14(2):7. doi: 10.1167/tvst.14.2.7 (PMC11806430; doi:10.1167/tvst.14.2.7)

Supplementary Figure 2. Fundus autofluorescence images of two study eyes with geographic atrophy (A-B), with the corresponding contours of areas graded as having definitely decreased autofluorescence by an external reading center (red; C-D).

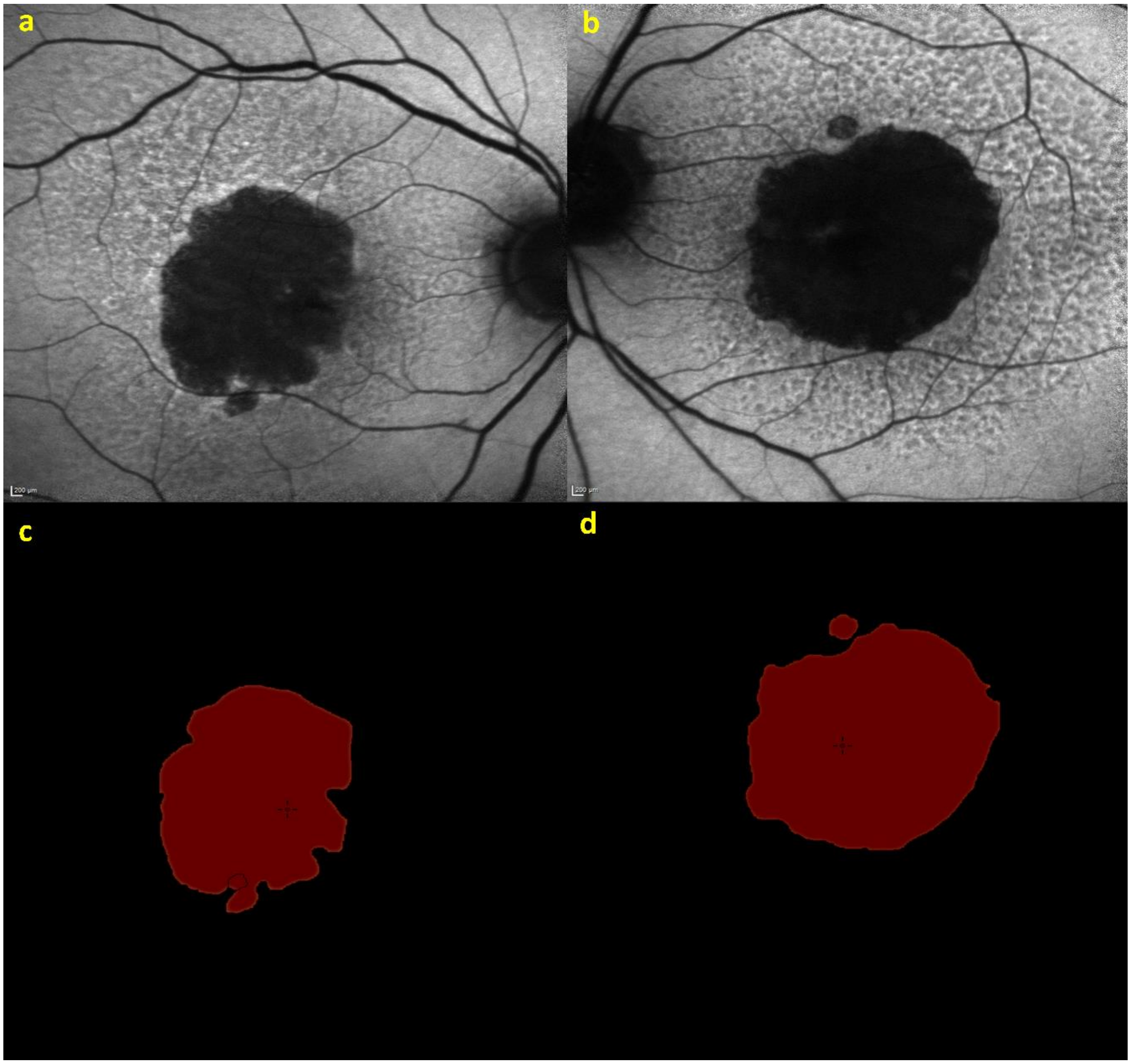

Supplement: Supplement 2 [file tvst-14-2-7_s002.pdf]
