# Supplementary material for: Structure–Function Relationships in Geographic Atrophy Based on Mesopic Microperimetry, Fundus Autofluorescence, and Optical Coherence Tomography
Source: Transl Vis Sci Technol. 2025 Feb 5;14(2):7. doi: 10.1167/tvst.14.2.7 (PMC11806430; doi:10.1167/tvst.14.2.7)

Supplementary Figure 3. Vertical optical coherence tomography (OCT) line scan of a study eye with geographic atrophy, with overlaying of microperimetry sensitivity measurements from the same visit at the corresponding retinal locations (red numbers in dB). Test loci with absolute scotomas are shown with “-1” (corresponding to the unfilled red squares in Figure 1). The same process was followed for horizontal OCT line scans.

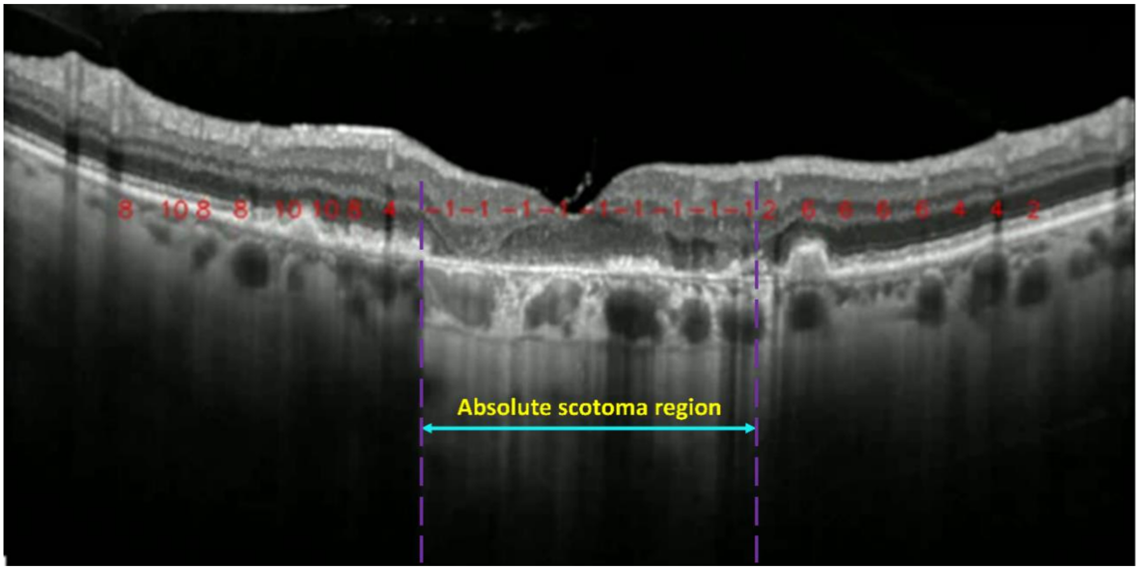

Supplement: Supplement 3 [file tvst-14-2-7_s003.pdf]
